# Neuroendocrine Disorders in Pediatric Craniopharyngioma Patients

**DOI:** 10.3390/jcm4030389

**Published:** 2015-03-09

**Authors:** Anna M. M. Daubenbüchel, Hermann L. Müller

**Affiliations:** 1 Department of Pediatrics, Klinikum Oldenburg, Medical Campus University Oldenburg, Rahel-Straus-Strasse 10, Oldenburg 26133, Germany; E-Mail: a.m.m.daubenbuechel@student.rug.nl; 2 University Medical Center Groningen, University of Groningen, Groningen 9713, The Netherlands

**Keywords:** craniopharyngioma, neuroendocrinology, neurosurgery, hypothalamic obesity, sleep, pituitary, neurocognitive, recurrence, quality of life, brain tumors, irradiation

## Abstract

Childhood-onset craniopharyngiomas are partly cystic embryonic malformations of the sellar/parasellar region. The therapy of choice in patients with favorable tumor localization is complete resection with a specific focus on maintaining optical and hypothalamic neuroendocrine functions. In patients with unfavorable tumor localization (*i.e.*, hypothalamic involvement), a limited hypothalamus-sparing surgical strategy followed by local irradiation is recommended. Involvement and/or surgical lesions of posterior hypothalamic areas cause major neuroendocrine sequelae. The overall survival rates are high (92%) but neuroendocrine disorders such as obesity and metabolic syndrome due to involvement and/or treatment-related hypothalamic lesions have major negative impact on survival and quality of life. Recurrences and progressions are frequent post-surgical events. Because irradiation is efficient in preventing tumor progression, appropriate timing of post-surgical irradiation is currently under investigation in a randomized multinational trial (KRANIOPHARYNGEOM 2007). Childhood-onset craniopharyngioma should be recognized as a chronic disease requiring treatment and constant monitoring of the clinical and quality of life consequences, frequently impaired due to neuroendocrine disorders, by experienced multidisciplinary teams in order to provide optimal care of surviving patients.

## 1. Introduction

Childhood-onset craniopharyngiomas (CP) are rare embryonic malformations of the sellar and parasellar area with low histological grade (WHO I°). Despite high survival rates (87%–95% in recent series), quality of life (QoL) is frequently impaired in long-term survivors due to mainly neuroendocrine sequelae caused by the anatomical proximity of the tumor to the optic nerve and to the hypothalamic-pituitary axes [1,2,3,4,5,6,7]. Any clinical significant improvement in the prognosis of CP patients will require the development of risk adapted neurosurgical and radiooncological treatment strategies in a multidisciplinary setting that provides medical as well as psychosocial support for these patients [8,9]. Due to the rareness of the disease, high survival rates, and adverse QoL effects, recent multicenter cooperation has already led to beneficial results in regard to long-term neuroendocrine deficits [2,10,11,12].

## 2. Epidemiology and Pathology

CP is a non-glial intracranial tumor derived from a malformation of embryonal tissue [13]. CP incidence is 0.5–2 cases per million persons per year, with 30%–50% of all cases presenting during childhood and adolescence [14,15]. In childhood and adolescence, its histological type is usually adamantinomatous with cyst formation [16,17]. More than 70% of the predominantly childhood adamantinomatous type of CP bear a mutation of the β-catenin gene [18]. Recently, a new mouse model of adamantinomatous childhood CP due to an activation of the Wnt signaling pathway was published [19]. 

## 3. Clinical Manifestations at the Time of Diagnosis

**Figure 1:**
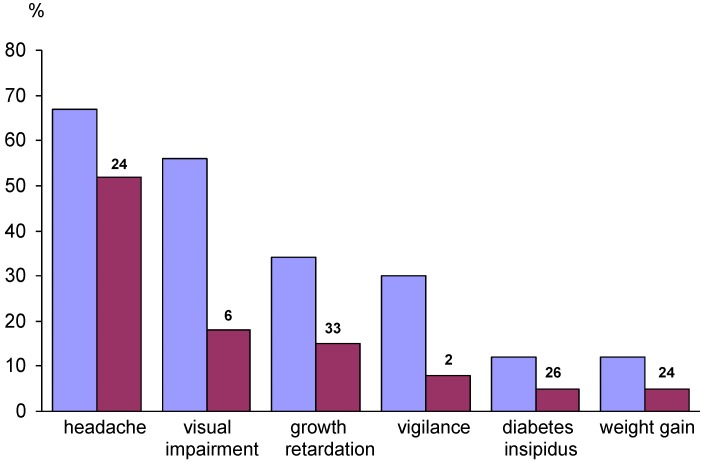
Manifestations before diagnosis of craniopharyngioma in children and adolescents. Frequency of occurrence of each manifestation before diagnosis (blue columns) and frequency of occurrence as the initial manifestation (red columns). The median time (months) from the appearance of each initial manifestation until diagnosis is indicated above each red column. In the overall group, the median time from the initial manifestation of disease until diagnosis was 12 months, with a range of 0.01–96 months. Modified from [20] with kind permission from Springer.

The diagnosis of CP is often made late—sometimes, years after the initial appearance of symptoms [20,21]—with the clinical picture at time of diagnosis often dominated by non-specific manifestations of intracranial pressure. Further primary manifestations are visual impairment (62%–84%) and endocrine deficits (52%–87%) (Figure 1). Endocrine deficits are frequently caused by disturbances to the hypothalamic-pituitary axes and affect growth hormone secretion (75%), gonadotropins (40%), adrenocorticotropic hormone (ACTH) (25%), and thyroid-stimulating hormone (TSH) (25%). At the time of diagnosis, 40%–87% of patients present with at least one hormonal deficit [4,22,23,24]. 

## 4. Imaging Studies

**Figure 2:**
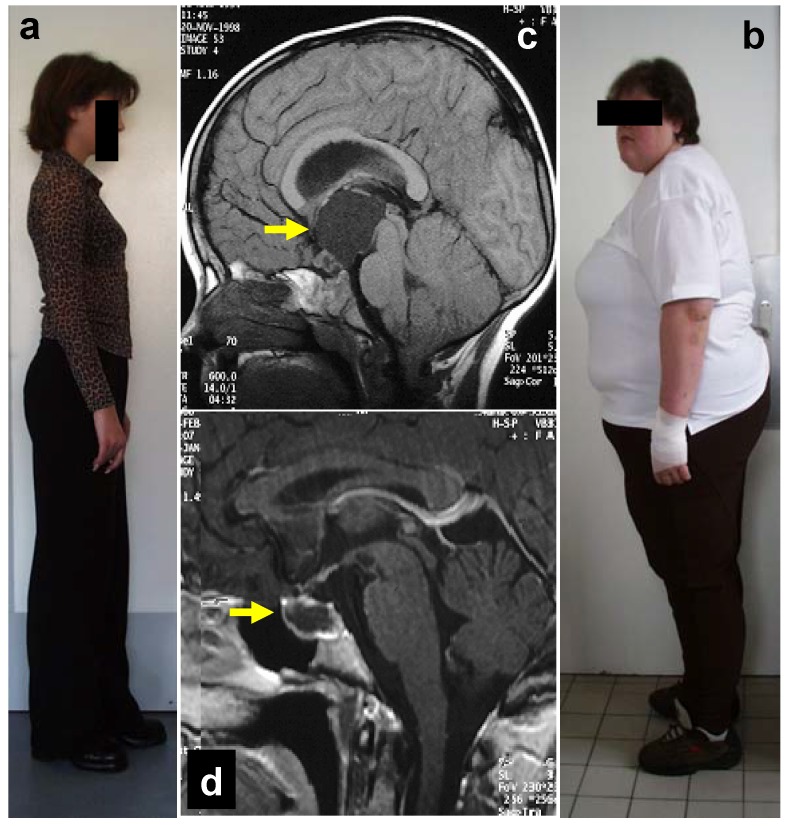
Degree of obesity in relation to the location of childhood-onset craniopharyngioma. In both patients, craniopharyngioma (as indicated by arrow on magnetic resonance imaging before surgery) could be completely resected. Both patients had complete hypopituitarism after surgery requiring endocrine substitution of all hypothalamic-pituitary axes. The patient depicted in (**B**) developed severe obesity due to hypothalamic lesions of suprasellar parts of craniopharyngioma (**C**). The patient depicted in (**A**) presented with a small tumor confined to the sellar region (**D**). After complete resection, she maintained a normal weight without any eating disorders. Modified from [20], with kind permission of Springer.

Both computerized tomography (CT) and magnetic resonance imaging (MRI) reveal that CP is typically a cystic tumor of the intra- and/or parasellar region. The most common location is suprasellar, with an intrasellar portion; only 20% of CPs are exclusively suprasellar and even less (5%) exclusively intrasellar [25,26]. CT is the only way to definitively detect or exclude calcifications in CP tissue, which is found in approximately 90% of these tumors (Figure 2). 

## 5. Treatment Strategies

### 5.1. Neurosurgery—Strategies and Effects

For favorably localized CP (*i.e.*, without involvement of hypothalamic or optical structures), the preferred treatment of choice is an attempt at complete surgical resection with preservation of visual and hypothalamic function [27,28,29]. For unfavorably located tumors too close to or too entangled with the optic nerve and/or hypothalamic structures, controversy exists over whether complete resection should still be attempted or whether a planned limited surgical resection should be performed [2,30,31]. Many authors take a critical view of planned radical resection in these cases because of the risk of surgically induced deficits (mainly hypothalamic) and the high rate of recurrence especially in infants and small children despite apparent complete resection [32,33]. Recurrences at ectopic location are reported [34]. Whereas following incomplete resection, the residual tumor shows progression in 71%–90% of patients, the progression rate after incomplete resection followed by radiotherapy is 21% [35]. Elowe-Gruau *et al.* [36] recently published their results of a single institution study at Necker, Paris, France, showing that a hypothalamus-sparing surgical strategy decreased the rate of severe long-term obesity in survivors without increasing their risk for local relapses when compared with a historical cohort treated before 2002 at the same experienced institution with a radical surgical approach [36].

However, the published literature to date [32,36,37,38,39,40,41,42,43] has not settled the controversy over the optimal treatment strategy for CP. Above all, effects of the chosen treatment sequence (progression-contingent irradiation of residual tumor *vs.* immediate irradiation) on QoL and neuroendocrine function are unclear based on the retrospective data published to date. 

**Figure 3:**
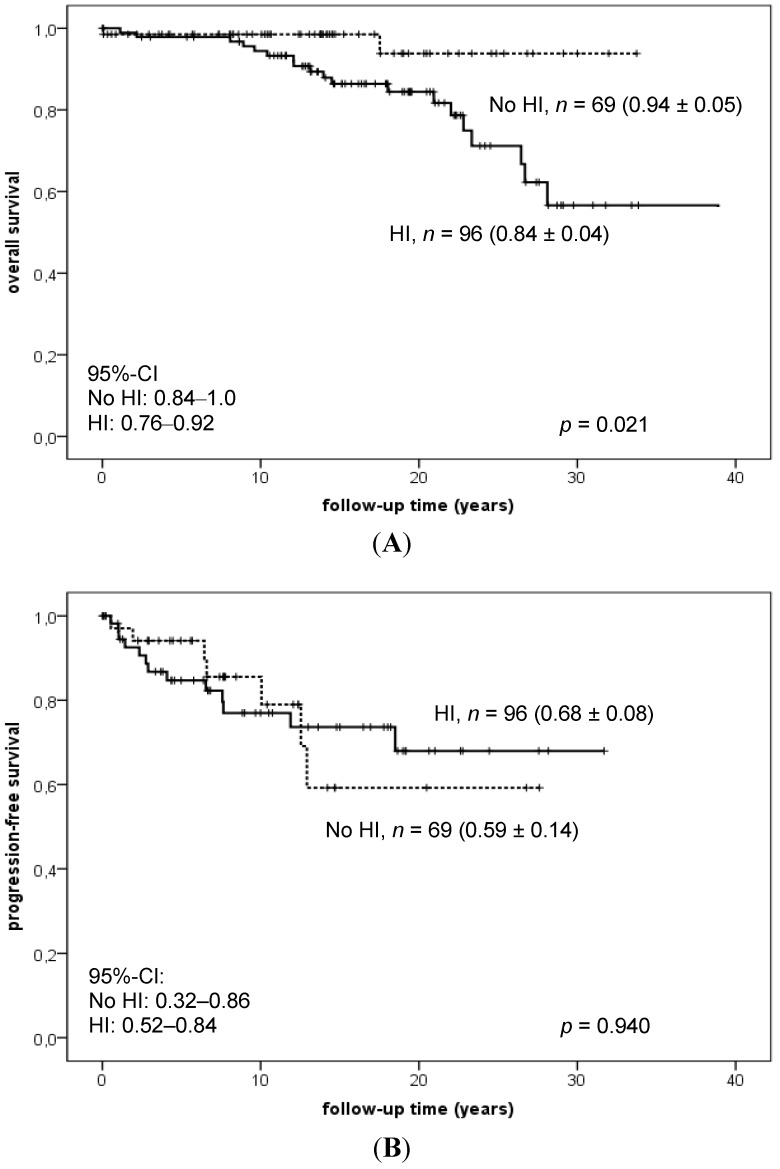
Kaplan-Meier analyses of overall (**A**) and progression-free survival rates (PFS) (**B**) depending on preoperative hypothalamic involvement in 177 patients with sellar masses (163 childhood-onset craniopharyngiomas, 14 cysts of Rathke’s pouch) recruited in the trial Hit Endo. Modified from [47] with kind permission from Bioscientifica.

Any discussion of treatment and follow-up strategies must take into account the neuroendocrine sequelae and QoL experienced by CP patients after treatment. Follow-up studies of QoL in children after complete resection of CP revealed that QoL depends on the experience of the operating neurosurgeon [44,45,46]. Daubenbüchel *et al.* [47] recently published that 20-year overall survival rates were significantly lower in CP patients with initial involvement of hypothalamic structures when compared with CP without hypothalamic involvement (Figure 3A,B).

### 5.2. Irradiation

A conventional, fractionated irradiation target (total) volume dose of 54 Gray has been established worldwide [35,48,49,50,51,52]. Clinical outcome data are still very limited for assessing the value of proton beam therapy compared to modern photon therapy, as the technique is available in only a few centers. However, proton beam therapy has the potential advantages of better conformation of dose to the target volume, sparing of critical structures, reduced integral dose, and lower dose of secondary neutrons, which should reduce the risk of secondary malignancies [53,54].

## 6. Neuroendocrine Sequelae

### 6.1. Pituitary Deficiencies

Pituitary hormone deficiencies are common in CP. At the time of diagnosis, 40%–87% of children [4,22,23,55] present with at least one hormonal deficit and 17%–27% [4,23,24] have been reported to have diabetes insipidus neurohormonalis. The rate of post-surgical pituitary hormone deficiencies increases due to the tumor’s proximity or even involvement of hypothalamic-pituitary axes [21,22,23,24,43,56,57,58]. Transient post-surgical diabetes insipidus occurs in up to 80%–100% of all cases [22,59]. The rate of permanent post-surgical diabetes insipidus ranges between 40% and 93% [22,23,24,37,43,46,58,59,60]. 

Growth hormone deficiency has been described at the time of diagnosis in 26%–75% of CP [4,60], and impaired growth may occur already years before diagnosis [21]. Growth hormone deficiency following treatment for CP is found in about 70%–92% of patients [21,46,61,62]. A positive response to growth hormone treatment is seen in most cases [63]. Normal growth in CP patients with proven growth hormone deficiency is reported in the literature [64]. In fact, CP patients with hypothalamic involvement were found to achieve normal adult height more often than those without hypothalamic involvement [21]. Even though this phenomenon of “growth without growth hormone” was described in CP almost five decades ago [65], the physiology of growth in these cases is still not fully understood—although insulin and/or leptin are suspected to play a compensating role in this phenomenon. 

### 6.2. Hypothalamic Dysfunction

Symptoms related to hypothalamic neuroendocrine dysfunction, such as obesity, behavioral changes, disturbed circadian rhythm and sleep irregularities, daytime sleepiness, and imbalances in regulation of body temperature, thirst, heart rate and/or blood pressure have been found at diagnosis in 35% of CP patients [24]. The rate of neuroendocrine hypothalamic dysfunction dramatically increases following radical surgical treatment; in some series up to 65%–80% [24,59]. Even though pre-surgical evaluation of hypothalamic damage is difficult both clinically and radiologically [56], tumor involvement of the third ventricle or obstructive hydrocephalus are suggestive findings [22]. A three level clinical grading system for hypothalamic dysfunction has been suggested based on the degree of obesity and hypothalamic tumor involvement [58].

**Figure 4:**
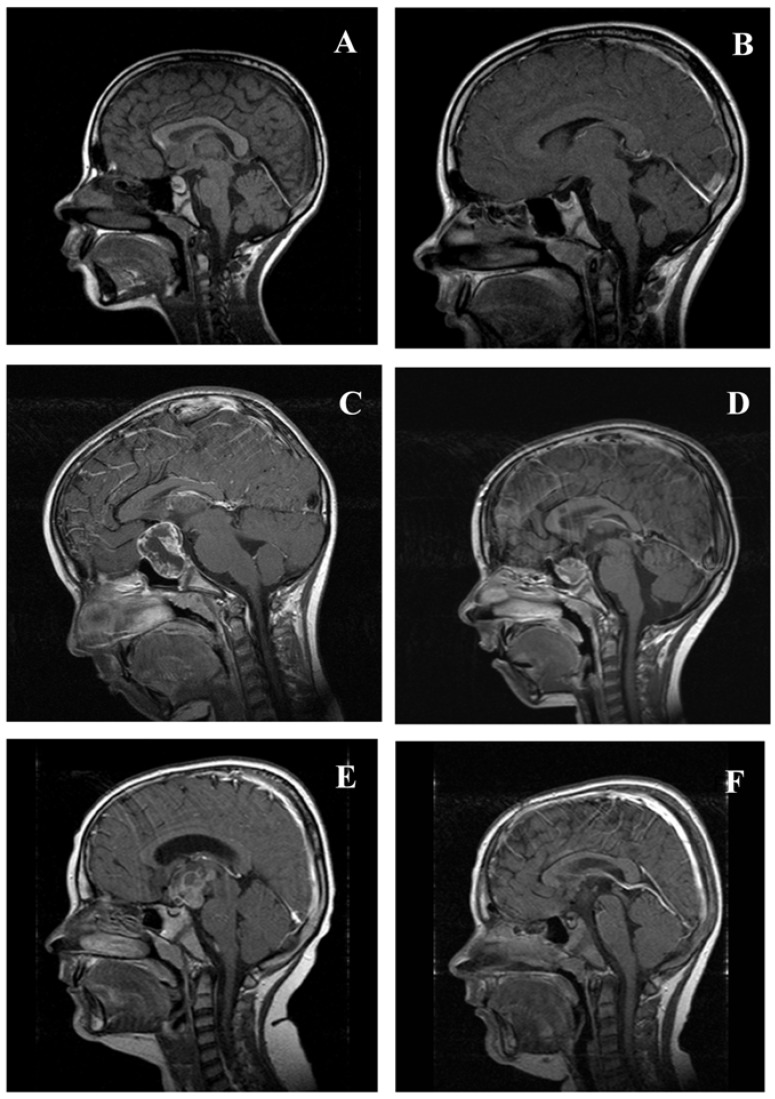
BMI and MRI imaging at diagnosis and 36 months after surgery in three cases of childhood-onset craniopharyngioma (CP) with different grades of hypothalamic involvement/lesion. (**A**,**B**) CP confined to the intrasellar space (0° no hypothalamic involvement (**A**)/surgical lesion (**B**)). BMI at diagnosis: −0.11 S.D.; BMI 36 months after complete resection: −0.41 S.D. (**C**,**D**) CP involving the anterior hypothalamus (I° hypothalamic involvement (**C**)/surgical lesion of the anterior hypothalamic area (**D**)). BMI at diagnosis: −1.75 S.D.; BMI 36 months after complete resection: −0.43 S.D. (**E**,**F**) CP involving the anterior and posterior hypothalamus (II° hypothalamic involvement (**E**)/surgical lesion of the anterior and posterior hypothalamic area (**F**)). BMI at diagnosis: +6.08 S.D.; BMI 36 months after complete resection: +6.79 S.D. Modified from [46], with kind permission from Bioscientifica.

Associated with high morbidity, suprachiasmatic CP with hypothalamic involvement are difficult to treat. Surgical removal of CP tissue beyond the mammillary bodies (*i.e.*, in the posterior hypothalamic area) endangers hypothalamic structures and may cause severe neuroendocrine deficits such as hypothalamic obesity [30,46]. With the aid of imaging studies, several reports have indicated that the degree of obesity of affected CP patients is positively correlated with the degree of hypothalamic damage [46,66,67,68]. Fjalldal *et al.* [69] recently published the results of a cross-sectional study of 42 patients who were analyzed for cognitive performance and psychosocial health at a median follow-up of 20 years after diagnosis of CP. The authors observed disturbed attention and impaired processing speed in adults with CP when compared with matched normal control. The deficits were most pronounced in patients with hypothalamic involvement of CP [69]. Taking these considerations into account, novel classifications of pre-surgical involvement and postsurgical lesions of hypothalamic structures based on MRI were recently published [28,46]. The classification might help to establish more risk adapted surgical strategies (Figure 4) based on a grading of pre-surgical hypothalamic involvement and post-surgical hypothalamic lesions.

### 6.3. Obesity and Eating Disorders

Rapid weight gain and severe obesity are the most perplexing neuroendocrine complications due to hypothalamic involvement and/or hypothalamic surgical lesions of CP. Weight gain in CP patients often occurs years before diagnosis [21], with 12%–19% of patients reported to be obese at diagnosis [4,23,59,60]. Weight gain occurs despite adequate endocrine replacement of pituitary hormone deficiencies. The hypothalamic disturbance in energy management contributes to the development of severe obesity and is exacerbated by factors limiting physical activity such as marked daytime sleepiness, disturbances of circadian rhythms, and neurological deficits [70]. The degree of obesity frequently increases early after treatment and rapid weight gain typically occurs during the first 6–12 months after treatment [60,67,71]. Following CP treatment, the prevalence of severe obesity is higher, reaching up to 55% [23,37,59,60,64,71,72,73,74]. Obesity and eating disorders result in increased risks of metabolic syndrome [64] and cardiovascular disease [68], including sudden death events [75], multisystem morbidity [76], and mortality [48,59,77,78,79,80,81,82,83].

**Figure 5:**
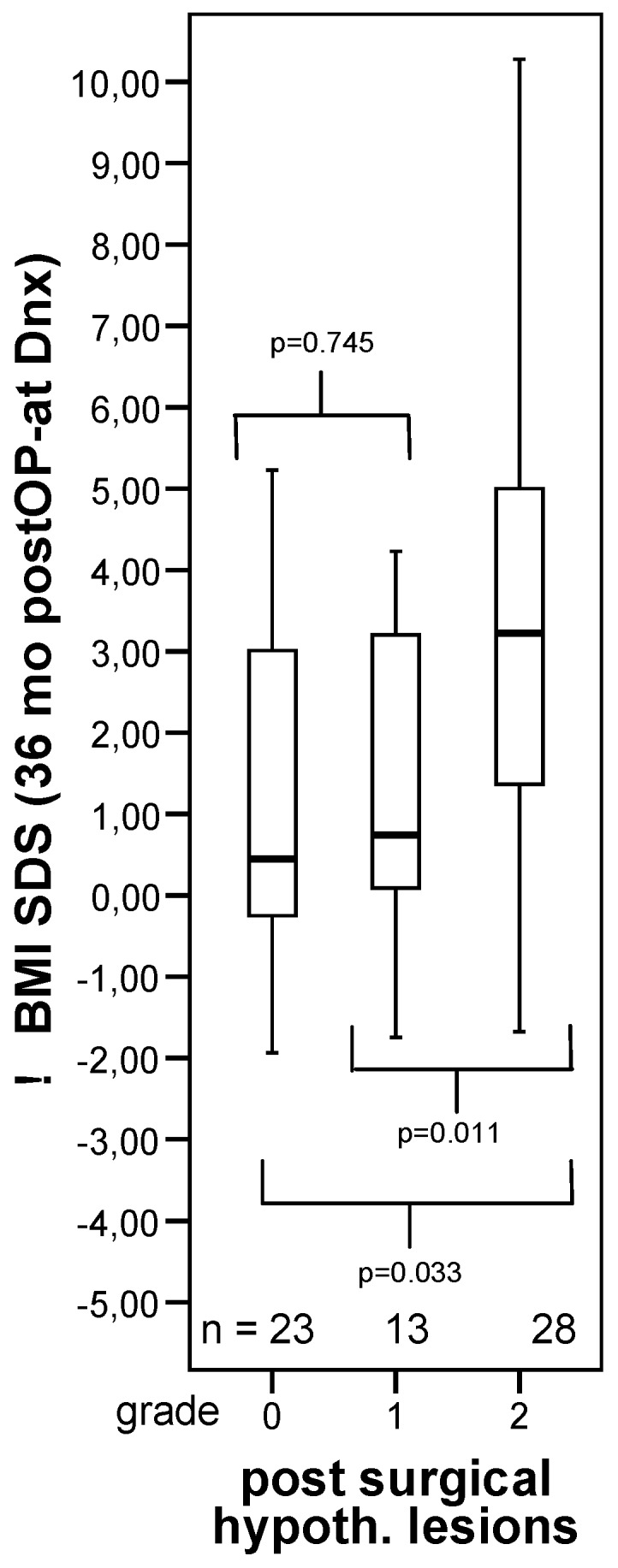
Changes in body mass index (BMI SDS) during first 36 months after diagnosis of 117 childhood-onset craniopharyngioma patients recruited inKRANIOPHARYNGEOM 2000 relative to the extent of surgical hypothalamic lesions (grade 0–2). The horizontal line in the middle of the box depicts the median. The edges of the box mark the 25th and 75th percentile. Whiskers indicate the range of values that fall within 1.5 box-lengths. Modified from [46], with kind permission from Bioscientifica.

Although the relation of severe obesity with hypothalamic lesions is obvious in CP patients [67,68,84], the neuroendocrine mechanisms responsible for increased prevalence of cardiometabolic complications in these patients are still unclear. It is likely that in case of suprasellar extension, hypothalamic neuroendocrine function will be compromised and will remain compromised to a certain extent when treated surgically or with irradiation. The hypothalamus plays a predominant role in keeping the internal environment stable by synchronizing biological clock mechanisms and circadian rhythms [85]. Recent data indicate that an adequate balance of the autonomic nervous system equilibrium is crucial for metabolism. It is well known that adipose tissue is richly innervated by sympathetic nerve fibers that control lipolysis. Consequently, it appears that lipogenesis is also controlled by parasympathetic innervation of adipose tissue originating from separate sympathetic and parasympathetic neurons in the periventricular nucleus and suprachiasmatic nucleus [86]. Such a high level of differentiation puts the suprachiasmatic nucleus in a key position to balance circadian activity of both branches of the autonomous nervous system. Considering the large proportion of CP patients with damage to suprasellar structures, it is likely that CP involving hypothalamic areas and/or the effects of treatment of these tumors damage the suprachiasmatic hypothalamic nucleus. This in turn affects the regulation of central clock mechanisms, which predisposes to neuroendocrine alterations in metabolism. Clearly, surgical strategies to preserve hypothalamic integrity are mandatory for the prevention of neuroendocrine deficits such as severe obesity owing to hypothalamic lesions (Figure 5).

When elevated serum leptin levels relative to body mass index were found in CP patients with a suprasellar tumor extension [87], researchers suggested that normal appetite inhibition failed to occur in these patients due to disruption of hypothalamic receptors that regulates negative feedback loops in which leptin, formed in adipocytes, binds to hypothalamic leptin receptors. However, a study involving self-assessment by nutritional diaries revealed that hypothalamic obesity also occurs in CP patients even when caloric intake is comparable to controls matched for body mass index [88].

### 6.4. Physical Activity and Energy Expenditure

An analysis of physical activity by accelerometric assessments showed that CP patients demonstrated a markedly lower level of physical activity than healthy controls matched for body mass index [88]. Markedly increased daytime sleepiness and disturbances of circadian rhythms have been demonstrated in patients with severely obese CP [70]. Daytime sleepiness and obesity in these patients were both correlated with low nocturnal and early morning melatonin concentrations in saliva. The proposed pathogenic mechanism involves impaired hypothalamic regulation of circadian melatonin secretion in CP extending to the suprasellar area. Initial experiences with oral melatonin substitution in CP patients were promising: melatonin levels normalized and physical activity and daytime sleepiness improved significantly [89]. However, data on long-term effects of melatonin substitution on weight development and daytime sleepiness have not yet been published.

Polysomnographic studies in patients with CP and severe daytime sleepiness have revealed sleeping patterns typical for hypersomnia and secondary narcolepsy, *i.e.*, frequent “sleep-onset REM phases” (SOREM) [72,90,91]. Medication with central stimulating agents (methylphenidate, modafinil) had a markedly beneficial effect on daytime sleepiness in these patients [90]. Secondary narcolepsy should be taken into consideration as a pathogenic factor in severely obese CP patients. Mason *et al.* [92] treated five pediatric patients with CP and severe hypothalamic obesity with the central stimulating agent dextroamphetamine for the purpose of weight reduction. Dextroamphetamine therapy stabilized patients’ body mass index. The patients’ parents reported noticeable improvements in their child’s physical alertness and activity. 

A decreased metabolic rate, in terms of both resting and total energy expenditure (TEE), has been suggested to contribute to weight gain in CP patients. Adults and pediatric patients with childhood-onset CP were found to have a lower resting-energy expenditure (REE) compared to controls [67,93,94] that was not explained by differences in terms of body composition. The energy intake/REE ratio was lower in those with tumors involving the third ventricle [67]. Impaired physical activity might be associated with an overall lowering of TEE [67,68,88,93]. Further factors potentially contributing to decreased physical activity are neurological and visual deficits, psychosocial difficulties, and increased daytime sleepiness.

### 6.5. Autonomous Nervous System

Lustig *et al.* [95,96] hypothesized that hypothalamic disinhibition of vagal output might be a cause of increased β-cell stimulation in CP patients, and that this disinhibition leads to hyperinsulinism and severe obesity. The authors therefore studied treatment with the somatostatin analogue octreotide, which suppresses β-cell activity [95]. 

Several reports [97,98] have hypothesized that decreased physical activity and severe obesity in CP patients could be related to impaired central sympathetic output. Roth *et al.* observed reduced urine concentrations of catecholamine metabolites correlating with the degree of obesity and the level of physical activity [99].

### 6.6. Appetite-Regulation

Roth *et al.* analyzed the gastrointestinal hormones ghrelin and peptide YY and their effect on satiety regulation in patients with CP and hypothalamic obesity [100]. Their findings support the hypothesis that reduced ghrelin secretion and impaired postprandial suppression of ghrelin in patients with CP and severe hypothalamic obesity results in disturbed regulation of appetite and satiety. Serum concentrations of peptide YY did not differ between normal weight, obese, and severely obese CP patients. A possible pathogenic role of peripheral α-melanocyte-stimulating hormone in CP obesity has also been reported [101].

Functional MRI was used in CP patients to examine the hypothesis that hypothalamic damage due to the tumor and/or its treatment results in enhanced perception of food reward and/or impaired central satiety processing [102]. Following a test meal, controls showed suppression of activation by food cues while CP patients showed trends towards higher activation in functional MRI. The authors conclude that perception of food cues may be altered in hypothalamic obesity, especially after eating, *i.e.*, in the satiated state. The functional MRI approach is encouraging for performing future mechanistic studies of the brain response to food cues and satiety in patients with hypothalamic obesity due to CP.

### 6.7. Treatment of Hypothalamic Obesity

Due to disturbances in energy expenditure, appetite-regulation, and central sympathetic output, CP patients with hypothalamic obesity typically develop morbid obesity that is mainly unresponsive to conventional lifestyle modifications (diet and exercise) [103]. Based on impairment of sympatho-adrenal activation manifesting as a reduced hormonal response to hypoglycemia, treating this disorder with amphetamine derivates has been suggested [104,105]. Use of dextroamphetamine started at 10 months post-surgical intervention for CP and lasting for 24 months was shown to diminish continuous weight gain and stabilize body mass index [92]; importantly, spontaneous physical activity increased significantly. Even shorter periods of dextroamphetamine treatment caused a subjective improvement in daytime sleepiness [106]. Elfers and Roth also observed beneficial effects of central stimulating agents on weight development in CP patients [107]. 

CP patients with hypothalamic obesity have a “parasympathetic predominance” of the autonomic nervous system induced by vagal activation and manifesting as daytime sleepiness, and reduced body temperature and heart rate [108]. Parasympathetic stimulation causes insulin secretion by way of direct activation of β cells as well as promotes adipogenesis. As insulin is an anabolic hormone, it has been suggested as an important driver of weight gain in hypothalamic obesity. Octreotide is a somatostatin analogue and thus causes reduction in insulin secretion. Lustig *et al.* used octreotide in a double-blind randomized controlled study in children with hypothalamic obesity and demonstrated moderate reductions in weight gain [95]. The authors showed that insulin levels during a proof-of-concept oral glucose tolerance test decreased without leading to major changes in glucose tolerance. This study was followed by a larger trial performed using octreotide LAR in 60 patients with cranial surgical interventions that led to hypothalamic obesity [109]. This 6-month intervention showed no efficacy in changing body mass index and the open label segment of this study was terminated earlier than planned due to an increased risk of gallstone formation. 

Initial experiences with bariatric surgery in severely obese CP patients achieved sufficient tolerability and short-term weight reduction [110,111,112]. An instant improvement of binge-eating behavior in patients with CP immediately after laparoscopic adjustable gastric banding (LAGB) was observed, but failed in long-term weight reduction. Nevertheless, weight stabilization could be achieved during regular follow-up monitoring [113]. 

In a systematic review and meta-analysis of the literature, Bretault *et al.* [114] analyzed the 12 months outcome after bariatric surgery for hypothalamic obesity due to CP and demonstrated that Roux-Y gastric-bypass, sleeve gastrectomy, and biliopancreatic diversion are the most efficient bariatric procedures for weight reduction in hypothalamic obesity of CP. However, treatment with invasive, non-reversible bariatric methods is controversial in the pediatric population because of medical, ethical, and legal considerations [113,115,116].

Despite the availability of the above-mentioned promising therapeutic approaches, it must be emphasized that currently no generally accepted (pharmacological or bariatric) therapy for hypothalamic obesity in CP has been shown to be effective in randomized studies [117]. 

### 6.8. Quality of Life, Neurocognitive Outcome and Psychosocial Functioning

QoL in CP patients can be affected by both the tumor itself and the treatment received. Reports assessing psychosocial and physical functioning show variable results, ranging from excellent in a majority of subjects to impaired function in almost half of the patients [23,59,118,119,120]. The most common areas of difficulty reported include emotional and social functioning, with CP patients rating their psychosocial status to be lower than their physical health [59]. Other challenges included somatic complaints such as pain, reduced mobility, and self-care [43,59]. Behavioral questionnaires indicate a high rate of psychopathological symptoms, including depression, anxiety, and withdrawal. The most frequent problems in CP patients’ everyday functioning include difficulties in learning, unsatisfactory peer relationships, inability to control emotions, and concerns regarding physical appearance and body image [62,121]. However, eating behavior and the rate of eating disorders are similar when compared with BMI-matched controls [122].

Factors associated with worsening quality of survival outcomes as well as neurocognitive and psychosocial functioning include preoperative functional impairment and younger age at diagnosis; furthermore tumor characteristics including hypothalamic and third ventricle involvement at presentation and larger tumor volume. Treatment strategies have also been implicated, with worse outcomes for surgery alone compared to limited surgery followed by irradiation and for multiple operations for tumor recurrence. Neuroendocrine, neurological, and ophthalmological sequelae all adversely affect QoL outcome [23,24,43,56,59,118,119]. Hypothalamic neuroendocrine deficits were found to have the most important negative impact on social functioning, physical ability, and body image [59,71,119].

Long-term neurocognitive complications following treatment for CP include cognitive problems, particularly those affecting episodic memory, executive function, attention, and working memory [59,62,121,123,124,125,126,127,128]. In a recent report, Özyurt *et al.* [129] provided first evidence that hypothalamic damage impacts on neural correlates of memory retrieval in medial prefrontal cortex, indicating a less efficient use of a prefrontal area involved in executive control processes. The authors propose that the deactivation failure in the patients’ anterior rostral medial prefrontal cortex is related to an increased coupling with the thalamus and reflects a reduced efficiency to flexibly adapt to task demands [129].

Furthermore, long-term survivors of CP treated primarily with subtotal surgical resection followed by irradiation were also found to have educational and psychological deficits [62]. Neurocognitive deficits include slower cognitive speed, memory disturbances, attention problems, and behavioral instability [62,121,123,125,126,128,130]. While intact intellectual functioning has been reported in up to 82% of patients, visual memory is impaired despite normal visual-spatial abilities [62,121]. The acquired deficits in higher cognitive processing such as attention problems are considered precursors to poor academic achievement. 

### 6.9. Survival and Late Mortality

Survival rates in CP patients are generally high [7]. However, disease related mortality can occur even many years after treatment. Data regarding survival includes primarily surgically treated patients. The reported post-surgical 5-year overall survival is 88%–94% [33,58,71,118], and the reported 10-year overall survival is 70%–92% [23,24,59,77,118], with a 20 year survival of 76%. Causes of late mortality include those directly related to the tumor or treatment such as chronic hypothalamic deficits, progressive disease with multiple recurrences, cerebrovascular disease, hormonal deficiencies, seizures, and non-alcoholic steatohepatitis leading to liver cirrhosis in some cases [24,56,58,59,77]. A recent review reports on substantial long-term morbidity with hypopituitarism, increased cardiovascular risk, hypothalamic neuroendocrine, visual and neurological deficits, reduced bone health, and reduction in QoL and cognitive function. The standardized overall mortality rate varies from 2.88–9.28 in cohort studies. Patients with CP have a 3–19-fold higher cardiovascular mortality in comparison to the general population. Women with CP have an even higher risk [131].

### 6.10. Treatment Strategies for Prevention of Neuroendocrine Sequelae

Despite high survival rates (87%–95%) in CP patients, research over the last several years reveals that long-term QoL is frequently impaired due to post-surgical late effects caused by the tumor’s close proximity to (or even anatomical entanglement with) optic structures and hypothalamic-pituitary axes. Radical surgery was the treatment of choice for several decades, with high reported rates of tumor control (65%–90%) in comparison with those obtained for incomplete resection (10%–50%) [42,132]. However, even at highly specialized and experienced surgical facilities, radical surgery can result in severe damage to the visual apparatus and hypothalamic-pituitary axes, behavioral disorders, and neurocognitive impairment [30]. 

Accordingly, management of CP remains controversial. Radical surgical strategies with the intention at gross-total resection frequently result in severe sequelae due to neuroendocrine hypothalamic deficits. Severe obesity is a major manifestation of hypothalamic syndrome and has negative impact on quality of survival [11]. On the other hand, hypothalamus-sparing surgery might increase the need for reoperation and/or irradiation [50]. 

An intracystic catheter insertion and subsequent instillation of substances inducing cyst shrinkage seems a beneficial strategy avoiding additional morbidity in cystic CP. Bartels *et al.* reported on outcome and neuroendocrine morbidity in six patients treated with cyst instillation of interferon alpha. None of the interferon-treated children suffered new endocrinological dysfunction, evidence of hypothalamic damage and none of them is obese [133,134].

Reports on long-term neuroendocrine outcome after “radiation therapy only” in CP are missing in the literature, presumably due to the rareness of “radiation therapy only”, and the necessity for long-term follow-up because of the delayed effects of irradiation on neuroendocrine function in such CP patients. 

**Figure 6:**
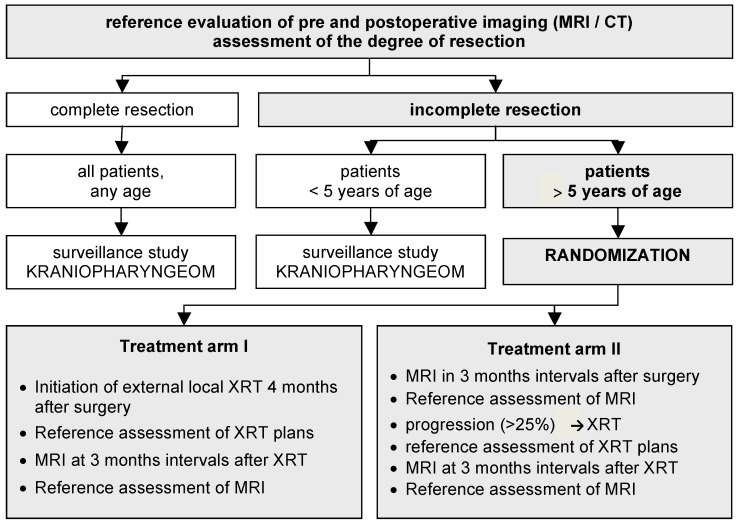
Study design of KRANIOPHARYNGEOM 2007 (www.kraniopharyngeom.net). Modified from [33], with kind permission from Thieme.

KRANIOPHARYNGEOM 2000, a large multicenter observational study that analyzed CP patients from Germany, Austria and Switzerland came to the conclusion that radical surgery is not an appropriate treatment strategy in CP patients with involvement of posterior hypothalamic structures [11,45,46]. KRANIOPHARYNGEOM 2007, a long-term study designed to analyze the appropriate time point of irradiation after incomplete resection, is currently focusing on patients’ QoL issues affected by the above-mentioned adverse effects (Figure 6).

Both studies also showed that the prognosis in long-term survivors is influenced by surgical expertise and explicitly recommend that surgical strategy be planned and performed by experienced surgeons, especially in light of the rarity of the disease and that treatment management of the tumors invading the hypothalamus has a strong influence on the prognosis of these patients [12,45,46]. A recent report of Elowe-Gruau *et al.* [36] analyzing two CP cohorts treated by the same highly experienced surgical team adds to the literature linking surgical experience and informed strategies to outcome results. These researchers are able to show that a limited surgery adapted to the degree of hypothalamic tumor involvement and highly respectful of hypothalamic structures, followed by adjuvant radiotherapy, does not lead to an increased rate of recurrence, decreases the risk of severe weight gain, and partially decreases the prevalence of endocrine deficiencies. Their findings support the efficacy and tolerability of irradiation as a salvage therapy in case of progressive residual CP. In their multivariate analysis, preoperative hypothalamic involvement and hypothalamus-sparing surgery independently predicted the presence of severe obesity at last follow-up [36]. In recent years, several grading systems of hypothalamic involvement have been published, which recommend hypothalamus-sparing surgical strategies in order to improve outcome and neuroendocrine sequelae in CP [13,28,36,45,46,69] (Table 1).

**Table 1:** Novel grading systems and treatment algorithms for childhood-onset craniopharyngioma patients based on magnetic resonance imaging. Modified from [7], with kind permission of Endocrine Press.

Author (Reference)	*n*	FU (year)	Grade 0 (0°)	Grade 1 (I°)	Grade 2 (II°)	Treatment Recommendation	Outcome Parameters
Puget [38]	65	3	no HI	HI (distortion/elevation) with negligible hypothalamic damage, the hypothalamus is still visible	tumor spread to the hypothalamus, which was no longer identifiable.	**0°**: gross-total resection (GTR) **I°**: attempt at GTR; if not achieved: 2nd surgery ± XRT **II°**: subtotal resection with hypothalamic preservation + XRT	lower BMI and similar relapse rate in a prospective cohort treated acc. to algorithm compared with historical cohort
Garre [13]	n.a.	n.a.	no HI	according to Puget *et al.* [38]	according to Puget *et al.* [38]	**0° + I°**: attempt at GTR by experienced surgeon; if not achieved: XRT **II°**: cyst drainage ± XRT (proton beam therapy at age <5 year)	n.a.
Müller [45,46]	120	3	no HI	HI/lesion of the anterior hypothalamus not involving the MB and the hypothalamic area beyond MB	HI/lesion of the anterior + posterior hypothalamic area, *i.e.*, involving the MB and the area beyond MB	**0°**: GTR **I°**: attempt at GTR; if not achieved: XRT **II°**: subtotal resection with hypothalamic preservation + XRT	higher BMI and lower QoL in the II° cohort treated by GTR resulting in posterior hypothalamic lesions
Flitsch [28]	n.a.	n.a.	no HI	according to Müller *et al.* [45,46], specifying sections below and above the diaphragm sellae	according to Müller *et al.* [45,46]	**0°**: GTR **I°**: attempt at GTR—transsphenoidal approach; if not achieved: XRT **II°**: subtotal resection with hypothalamic preservation—transcranial approach, followed by XRT	n.a.
Fjalldall [69]	42	20	no HI	suprasellar growth, not towards or into the 3rd ventricle (non-TGTV)	suprasellar growth towards or into the 3rd ventricle (TGTV)	**Non-TGTV**: GTR **TGTV**: subtotal resection with hypothalamic preservation + XRT	Lower cognitive performance in TGTV patients treated by GTR

Optimal care of CP is best provided in a multidisciplinary collaborative environment that includes experienced pituitary practitioners in not only neurosurgery and endocrinology, but also in radiation oncology, medical oncology, neuroophthalmology, neuroradiology, and neuropathology. McLaughlin *et al.* [135] provided the background and rationale for recognizing pituitary “centers of excellence” and suggested a voluntary verification process. Hoffmann *et al.* [12] recently analyzed changes in surgical strategy during the last 15 years by comparing a CP cohort treated between 2000 and 2006 with a more recent cohort treated between 2007 and 2013. The authors observed a change towards less aggressive surgical strategies (gross-total resections) during the last 15 years in childhood CP. 

## 7. Conclusions

Risk-adapted surgical strategies at initial diagnosis of CP should aim at a maximal degree of resection keenly focused on respecting the integrity of optical and hypothalamic structures to prevent severe neuroendocrine deficits and therein minimize consequences that could negative impact patient QoL. In case of hypothalamic involvement, hypothalamus-sparing surgical strategies are recommended in order to prevent hypothalamic damage and associated severe neuroendocrine sequelae. Local irradiation of residual tumor is efficient in preventing tumor progression. Because initial hypothalamic tumor involvement, especially of posterior hypothalamic structures, has an *a priori* effect on the clinical course [21,46], CP should be recognized as a chronic disease requiring constant monitoring of the consequences and medical resources for treatment in order to provide not only optimal QoL for patients, but also to garner additional information with the intent of minimizing what at present are severe consequences of both the disease and its treatment. CP patients should be treated by specialized and experienced multidisciplinary teams [11,30,136]. 
